# Supplementary material for: Influence of CT Image Matrix Size and Kernel Type on the Assessment of HRCT in Patients with SSC-ILD
Source: Diagnostics (Basel). 2022 Jul 8;12(7):1662. doi: 10.3390/diagnostics12071662 (PMC9321522; doi:10.3390/diagnostics12071662)
Supplement: Supplementary file 1 [file diagnostics-12-01662-s001.zip › diagnostics-1766217-supplementary.pdf]

Supplementary materials (violin plots)

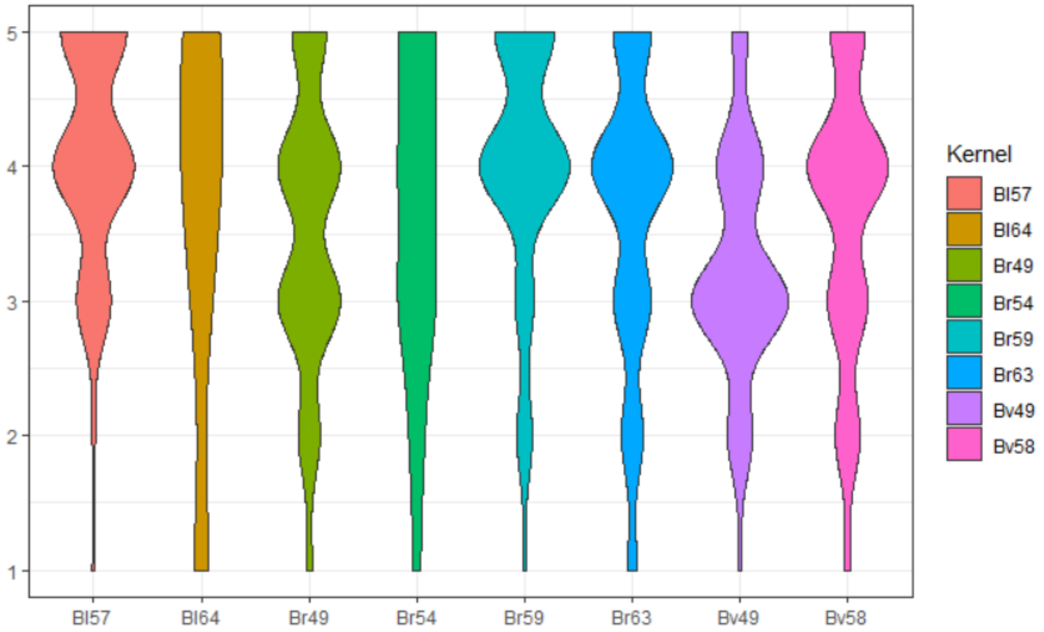

Figure S1. Sharpness per kernel, violin plot with the score distribution (Y-axis: 5-point Likert scale, X-axis: Kernel)

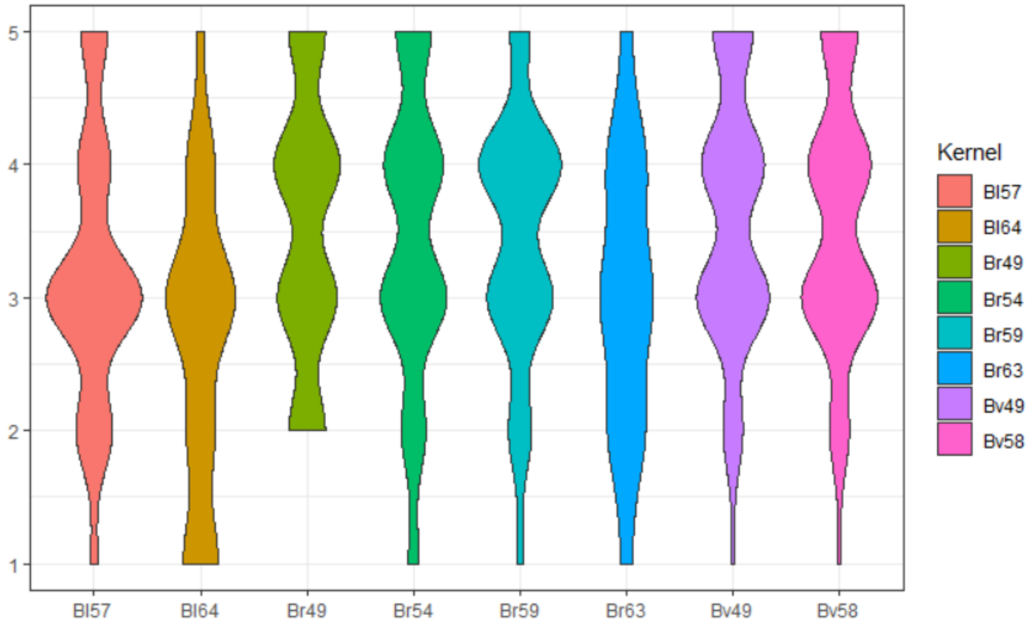

Figure S2. Noise per kernel, violin plot with the score distribution (Y-axis: 5-point Likert scale, X-axis: Kernel)

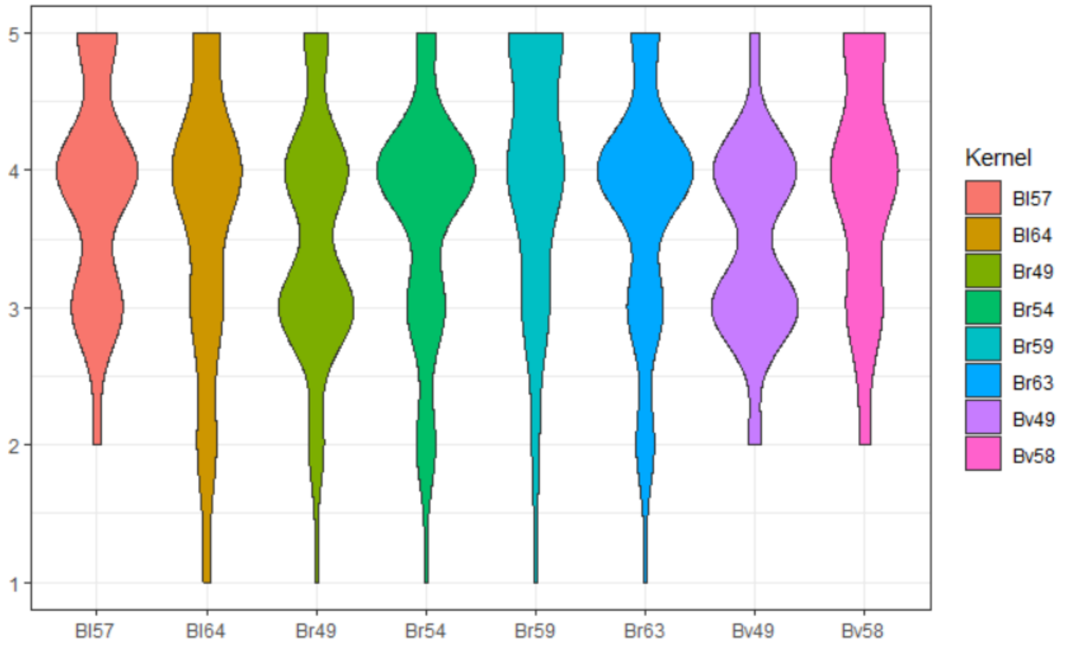

Figure S3. Detection of pathologies per kernel, violin plot with the score distribution (Y-axis: 5-point Likert scale, X-axis: Kernel)

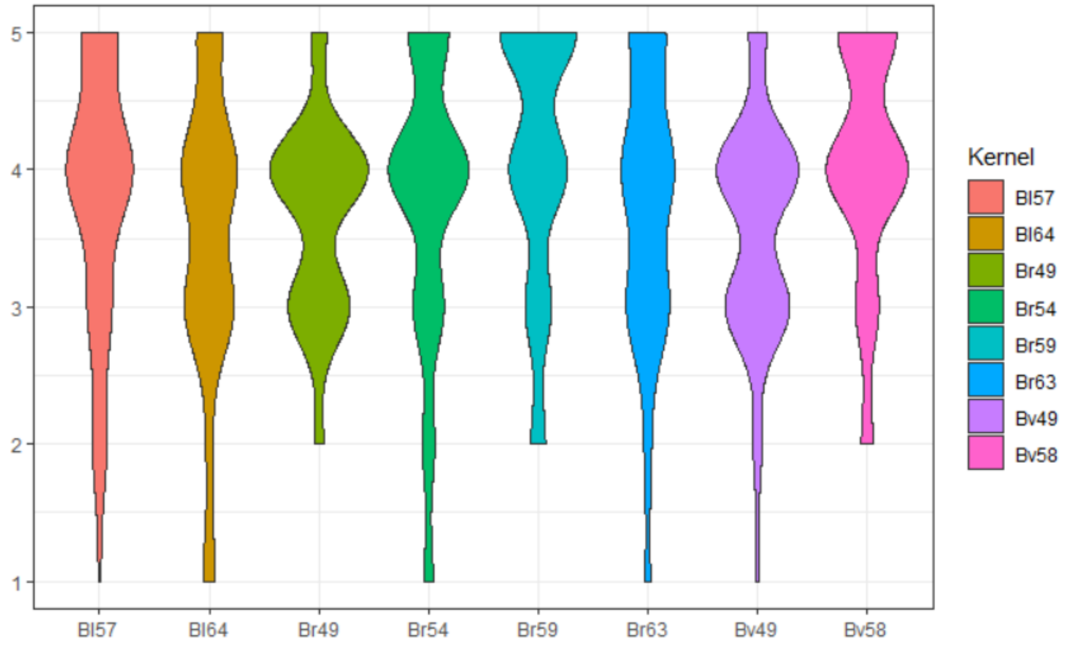

Figure S4. Depiction of bronchiole per kernel, violin plot with the score distribution (Y-axis: 5-point Likert scale, X-axis: Kernel)

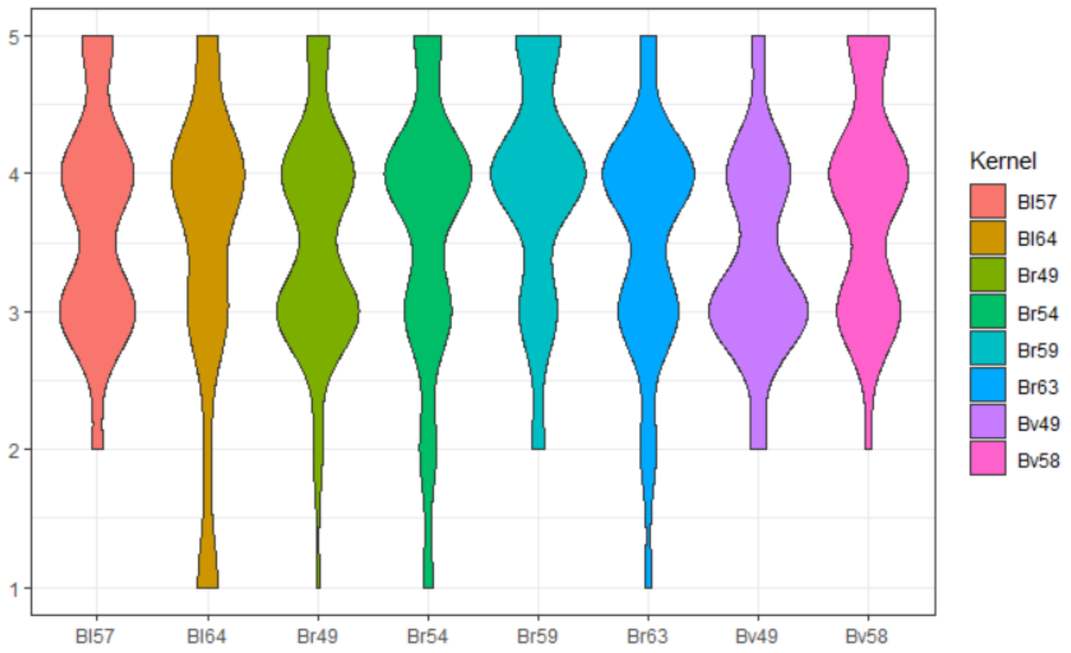

Figure S5. Overall image impression per kernel, violin plot with the score distribution (Y-axis: 5-point Likert scale, X-axis: Kernel)

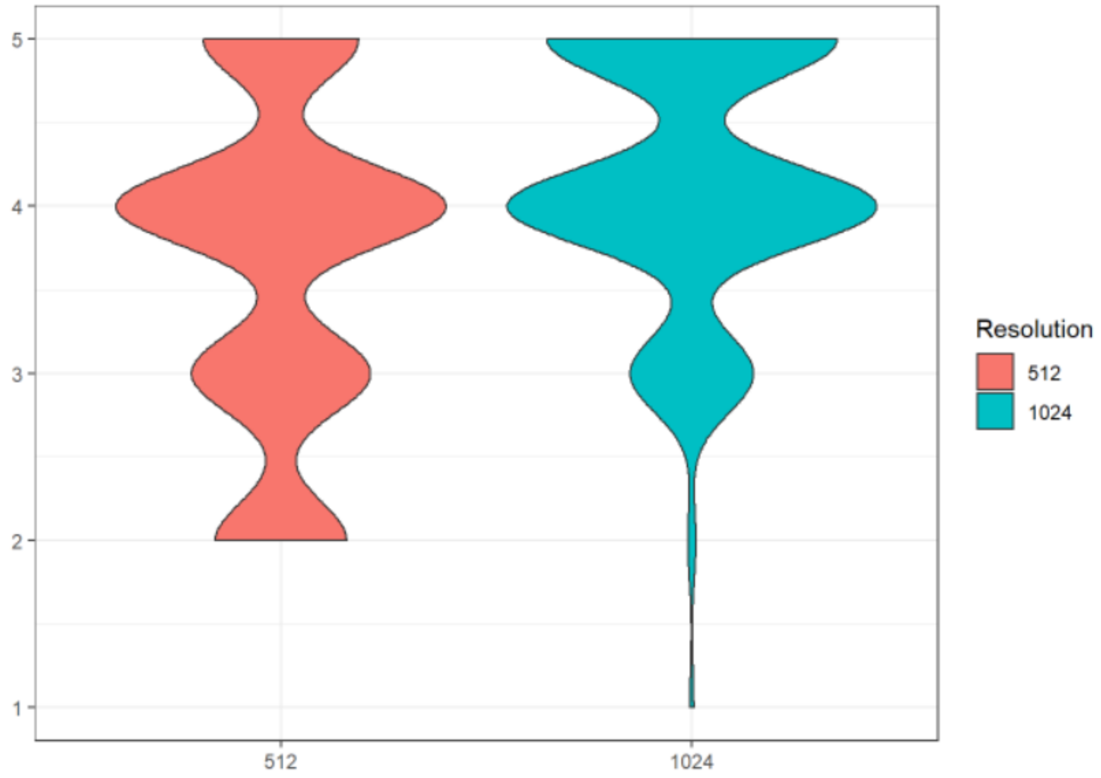

Figure S6. Sharpness per matrix resolution, violin plot with the score distribution (Y-axis: 5-point Likert scale, X-axis: Matrix resolution)

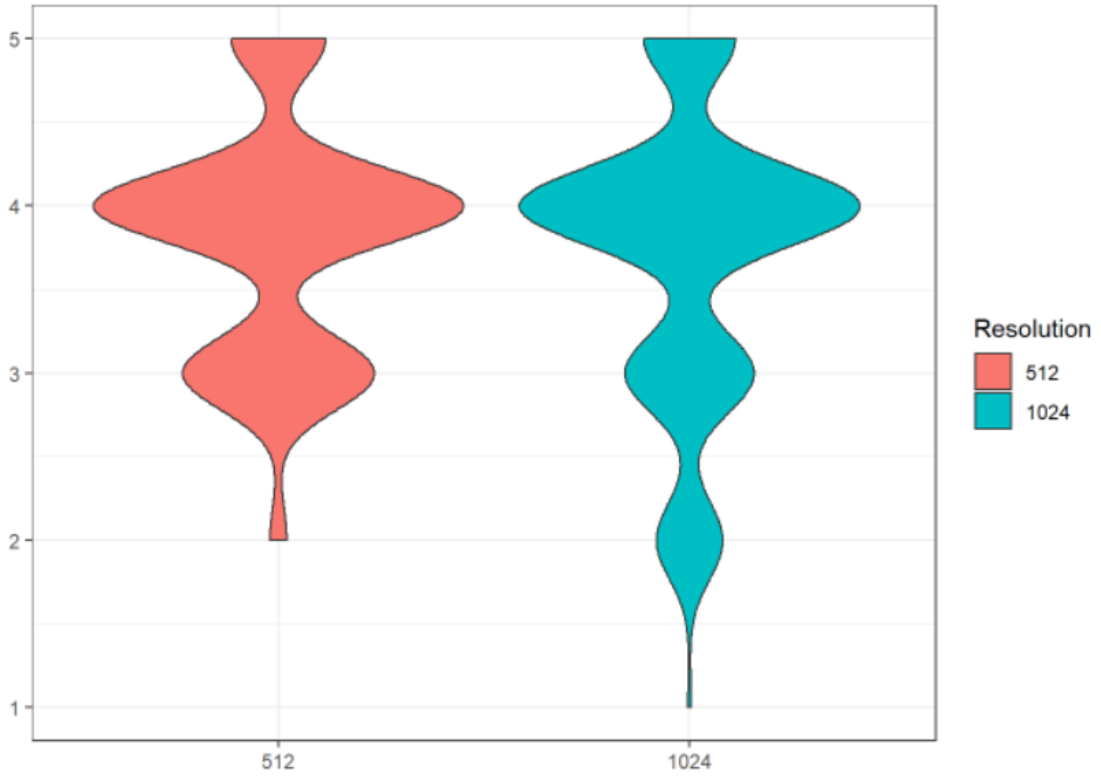

Figure S7. Noise per matrix resolution, violin plot with the score distribution (Y-axis: 5-point Likert scale, X-axis: Matrix resolution)

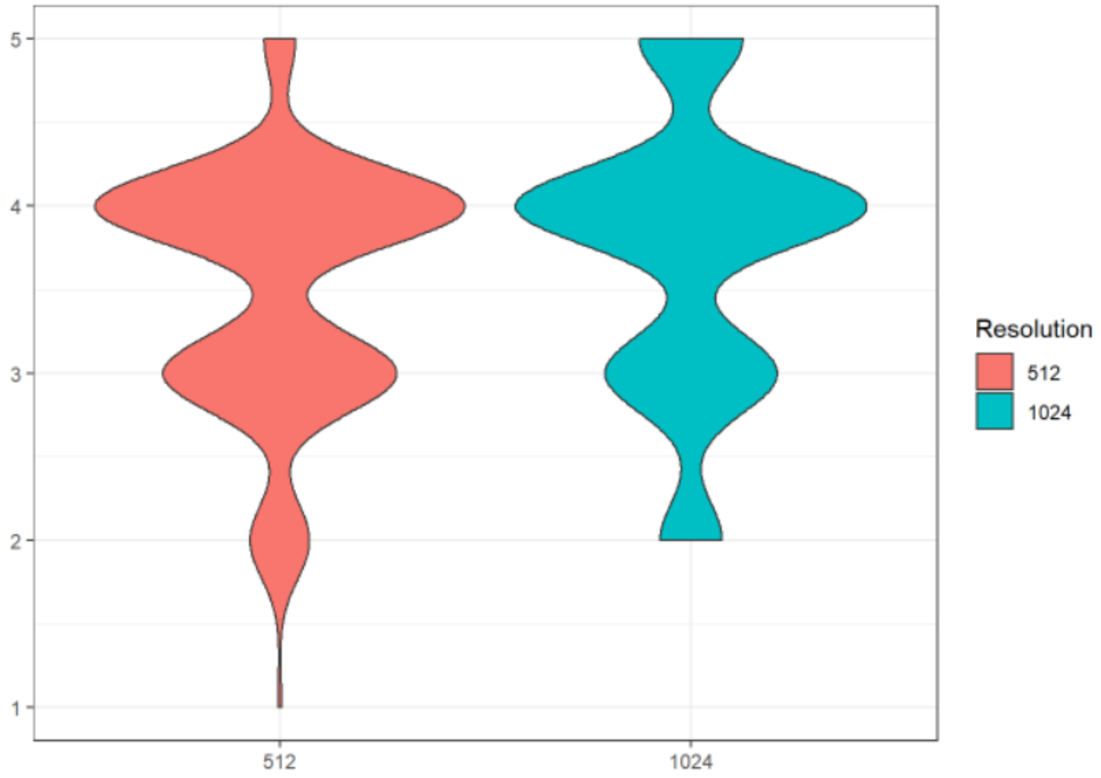

Figure S8. Depiction of bronchiole per matrix resolution, violin plot with the score distribution (Y-axis: 5-point Likert scale, X-axis: Matrix resolution)

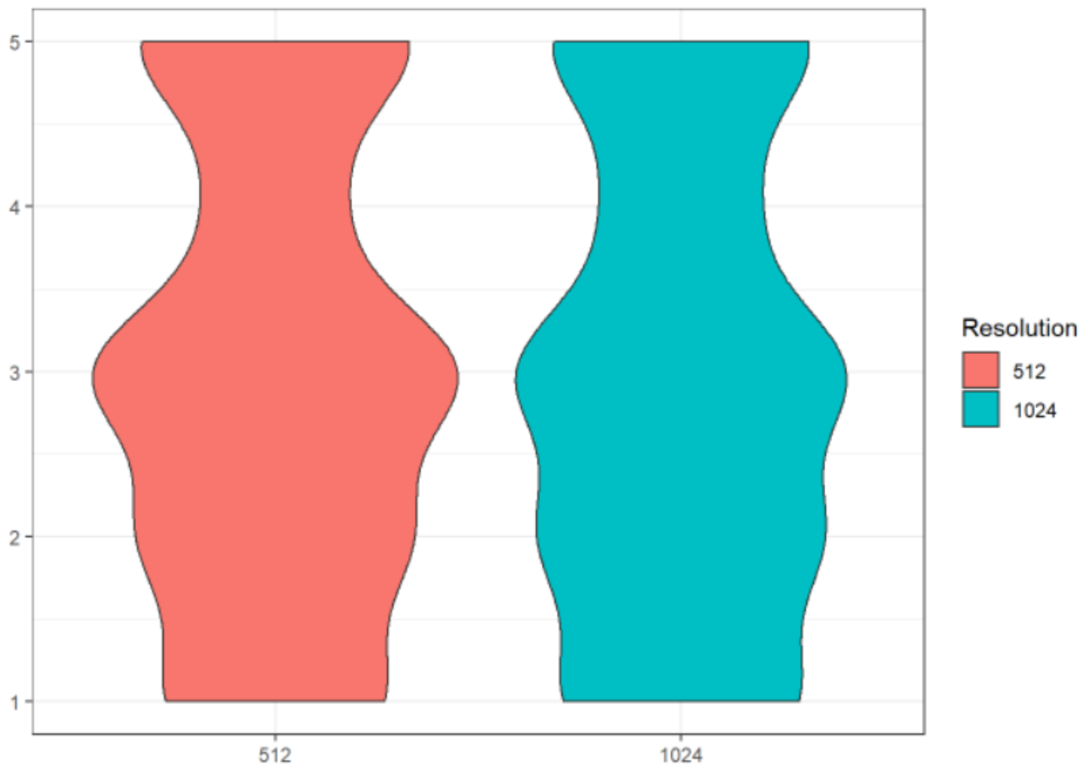

Figure S9. Extent of reticulations/bronchiectasis/fibrosis per matrix resolution, violin plot with the score distribution (Y-axis: 5-point Likert scale, X-axis: Matrix resolution)

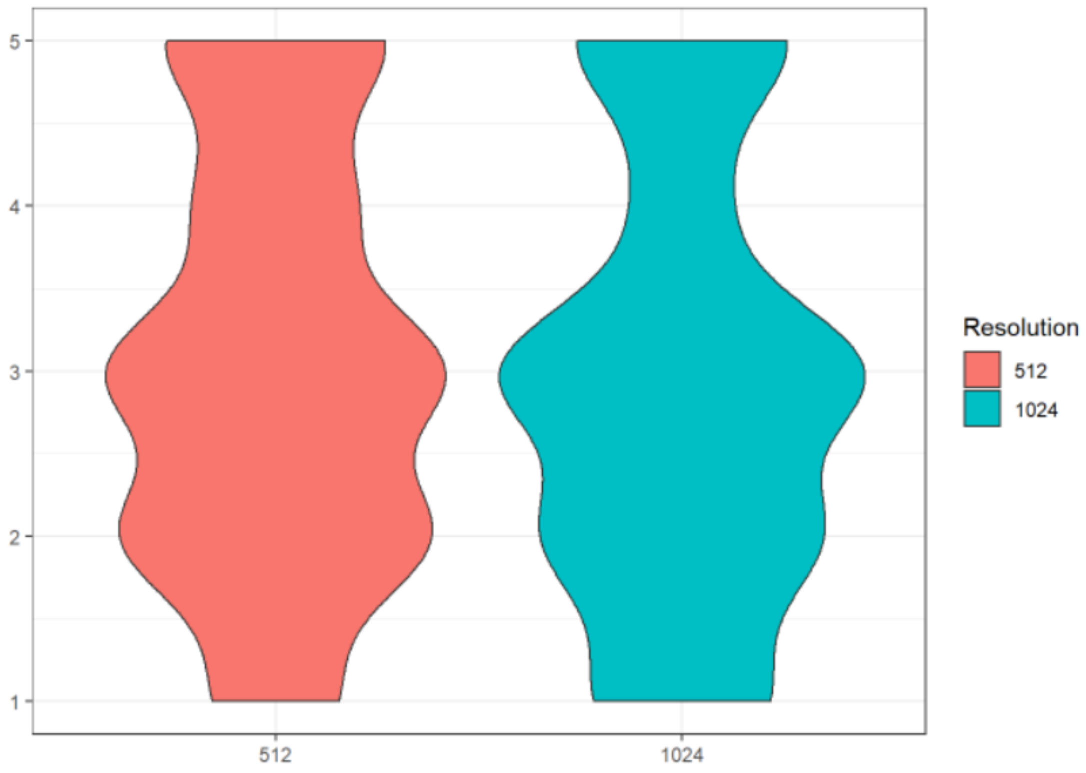

Figure S10. Extent of ground-glass opacities per matrix resolution, violin plot with the score distribution (Y-axis: 5-point Likert scale, X-axis: Matrix resolution)

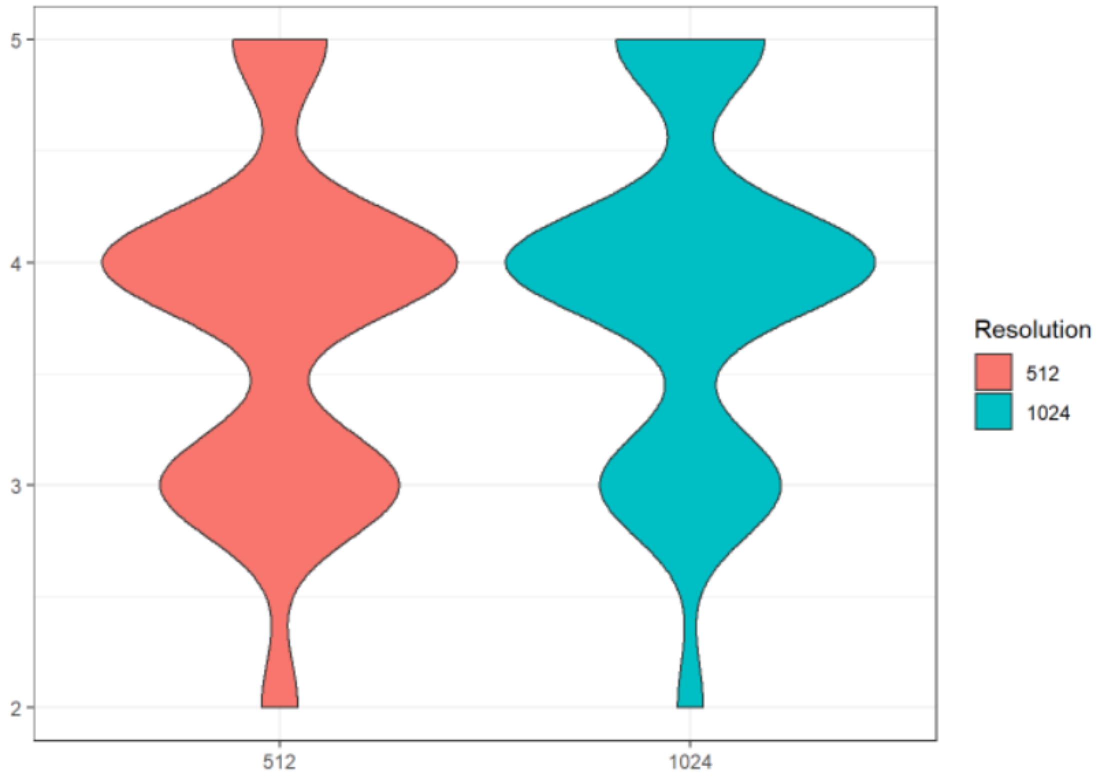

Figure S11. Overall image impression per matrix resolution, violin plot with the score distribution (Y-axis: 5-point Likert scale, X-axis: Matrix resolution)
